# Idiosyncrasy in Relation to Doses

**Published:** 1903-12-19

**Authors:** 


### IDIOSYNCRASY IN RELATION TO DOSES.

Recognised authorities on the art of therapeutics are all accustomed to draw attention to certain remedies as prone to vary widely in the degree of their action in different individuals. This is true in particular of drugs such as opium, arsenic, and belladonna, which produce, when given in excess, decidedly troublesome symptoms. These, and some few other remedies, it is usual to find catalogued as substances which are conspicuously influenced in the measure of their operation by the personal idiosyncrasy of the patient. They are distinguished as exceptional in this respect, whilst the great majority of remedies, it is implied, conform to a general rule of constancy and uniformity in the degree of their influence. This, if true, is, it must be admitted, somewhat extraordinary. On *a priori* grounds it would surely be expected that if the personal peculiarity of the patient influences the activity and operation of any one drug, it will, to a greater or less extent, influence the degree of action of all drugs. Though the remedy is varied, the element of personal peculiarity remains. Experience confirms this argument, and shows that not merely a few, but all remedies, are modified in the degree of their activity by that intangible but none the less real something which makes each man different from his fellows and gives to him the standing of a distinct individual. Idiosyncrasy, in a word, is not an exception but a rule, and the commonly imputed instances of its operation are merely extreme illustrations of a general principle that happen to cause considerable personal inconvenience. This doctrine has an important and direct application to the art of treatment. It implies that the doses of remedies in all instances require adjustment to the requirements of each individual case, and that one of these requirements is determined by personal idiosyncrasy. The action of any remedy is in reality the result of a reaction between the remedy administered and the tissues of the patient. The one may be weighed and measured by anyone capable of using a balance, but observation and experience are required to estimate and determine the range of influence of the other. Hence it is that treatment offers so large an opportunity for the cultivation of insight and judgment, and the more successfully these are developed the more sure will be the efficacy of the practitioner's prescriptions. There are certain influences which tend to prevent development in this direction. First there is a failure to recognise the truth of the principle above defined. Idiosyncrasy is attached as a modifying force to the action of a few selected remedies. It ought to be proclaimed as a principle in operation over the whole of the therapeutic area. In the second place, there is the almost universal practice of the text books to fix for each remedy a maximum and minimum dose. These are usually made to correspond to the doses quoted in the British Pharmacopœia. They are committed to memory when the student lives under the shadow of examinations and thus are found not infrequently to fix a habit for the practitioner. It is of course essential that the student shall have some rules by which to guide his early efforts, but such rules in no sense limit the freedom of the practitioner. The Pharmacopœia provides a list of doses, certainly not for the compulsion, hardly even for the guidance, of the physician. The advantage of such a list is, that it places the dispenser in a position to take note of any overdose which may by mistake be ordered in the prescription. Hence it tends to prevent accidental poisoning. But the practitioner is perfectly free to depart, and will often be well advised to depart, from the official quantities. He must, of course, accept responsibility for his decisions. But this he may readily do when he can rest with confidence on his own experience. Here is a guide immeasurably superior to the dogmatic directions of any artificial authority. The latter have their day and purpose. But the time comes for each man to stand securely on his own judgment. It is wise and necessary that in the days of pupilage insistence should be placed on the dangers of doses in excess. But there is at least an equal danger in the opposite direction. It is not a hazardous proposition to suggest that failure in treatment is more frequently due to a want of courage to prescribe remedies in due amounts than to defective judgment in the selection of the remedy. The dread of the official maximum causes the prescriber to hold his hand when he is possibly on the very eve of success. That maximum, we repeat, is not intended to be, and it ought not to be, a measure of the practitioner's freedom. Granted that he proceeds with due caution, he is fully justified in carrying his prescription to any lengths which the exigencies of the case may demand. How often, for example, in syphilis of the central nervous system is the administration of potassium iodide in 10 or 20 grain doses a complete failure, when a dose of three or four times these amounts proves a signal success. The principle holds good universally, and were it more widely recognised the virtues of drug treatment would command an ampler faith. Another influence which opposes the adequate study of the doses of drugs in relation to the requirements of the individual patient is the unceasing procession of new remedies. To try what is new rather than to study what is established is the ambition of the hour. Hence, whilst we have a "bowing acquaintance" with many drugs we are really intimate with none. The older school used fewer remedies. But they used them with confidence because they used them with knowledge. Therapeutic efficiency would be promoted by some return in this direction as well as by a more general recognition of the principle that doses are to be determined, not by an arbitrary standard, but by the requirements and capacities of the individual patient.

